# Mesenchymal Stem Cell‐Derived Exosomes: A Promising Therapeutic Strategy for Age‐Related Diseases

**DOI:** 10.1111/cpr.13795

**Published:** 2024-12-20

**Authors:** Bohua Wei, Mengting Wei, Haonan Huang, Ting Fan, Zhichang Zhang, Xiaoyu Song

**Affiliations:** ^1^ School of Pharmacy China Medical University Shenyang Liaoning Province China; ^2^ School of Stomatology China Medical University Shenyang Liaoning Province China; ^3^ China Medical University Shenyang Liaoning Province China; ^4^ Department of Computer, School of Intelligent Medicine China Medical University Shenyang Liaoning Province China; ^5^ The College of Basic Medical Science, Health Sciences Institute China Medical University Shenyang Liaoning Province China

**Keywords:** exosome, immunomodulatory, mesenchymal stem cell, senescence, tissue regeneration

## Abstract

The global increase in the aging population has led to a concurrent rise in the incidence of age‐related diseases, posing substantial challenges to healthcare systems and affecting the well‐being of the elderly. Identifying and securing effective treatments has become an urgent priority. In this context, mesenchymal stem cell‐derived exosomes (MSC‐Exos) have emerged as a promising and innovative modality in the field of anti‐aging medicine, offering a multifaceted therapeutic approach. MSC‐Exos demonstrate significant potential due to their immunomodulatory and anti‐inflammatory properties, their ability to inhibit oxidative stress, and their reparative effects on senescent tissues. These attributes make them valuable in combating a range of conditions associated with aging, such as cardiovascular diseases, neurodegeneration, skin aging, and osteoarthritis. The integration of exosomes with membrane‐penetrating peptides introduces a novel strategy for the delivery of biomolecules, surmounting traditional cellular barriers and enhancing therapeutic efficacy. This review provides a comprehensive synthesis of the current understanding of MSC‐Exos, underscoring their role as a novel and potent therapeutic strategy against the intricate challenges of age‐related diseases.

## Graphical abstract

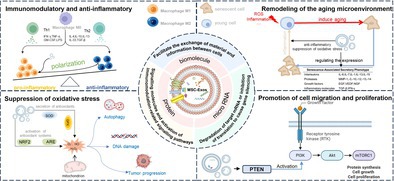

## 1 Introduction

As the global population ages, the morbidity and mortality rates of age‐related diseases are increasing, posing a great challenge to the public health system. Aging is a complex biological process involving multiple cellular and molecular mechanisms, including DNA damage, oxidative stress, cellular senescence, and tissue dysfunction [1]. Together, these processes lead to the development of a variety of diseases, such as cardiovascular and neurodegenerative diseases, which pose a serious threat to human health and quality of life. Therefore, exploring and researching therapeutic approaches for these diseases has become a top priority. In recent years, mesenchymal stem cells (MSCs) and their exosomes have attracted much attention in anti‐aging therapy, providing new insights and avenues for treating age‐related diseases.

MSCs are characterised as a population of multipotent stromal progenitor cells that have the ability to adhere to plastic and exhibit fibroblast‐like, express surface antigens, including CD105, CD90, and CD73, and differentiate into osteoblasts, adipocytes, and chondroblasts in vitro [2, 3]. As with other stem cells, MSCs exhibit self‐renewal and robust proliferative capabilities [4, 5]. However, recent studies indicate that the therapeutic efficacy of MSCs is not solely attributed to their pluripotency, and relying exclusively on their differentiation into tissues or organs for repair or therapy is currently impractical [6]. In contrast, their primary therapeutic role appears to be mediated through the paracrine release of bioactive molecules that modulate the microenvironment and activate endogenous tissue‐specific stem cells [7, 8, 9]. Notably, exosomes are pivotal in mediating the paracrine effects of MSCs, acting as intercellular messengers that transport bioactive molecules from MSCs to target damaged cells, thereby promoting cell survival, proliferation, and tissue repair, and holding promise for clinical applications [8, 9].

Exosomes, minute extracellular vesicles with diameters spanning from 30 to 150 nm [10], encapsulate a diverse cargo comprising proteins such as tetraspanins and heat shock proteins, lipids including sphingomyelin and cholesterol, as well as genetic material and small molecule metabolites that reflect the unique signature of the donor cells [11, 12]. These exosomes can deliver their bioactive cargo to target cells through various mechanisms, including fusion, endocytosis, and membrane fusion, thereby influencing the physiological functions of the recipient cells [13]. In recent years, the application of mesenchymal stem cell‐derived exosomes (MSC‐Exos) in anti‐aging research has garnered significant attention. For instance, several studies have demonstrated that MSC‐Exos can effectively decelerate skin aging, stimulate fibroblast proliferation and collagen synthesis, and mitigate inflammatory responses [14]. Moreover, MSC‐Exos plays a pivotal role in regulating cardiomyocyte function and promoting tissue repair post‐injury, which holds immense potential in treating age‐related cardiovascular diseases [15]. These findings not only strengthen the experimental foundation for the utilisation of MSC‐Exos in anti‐aging applications but also pave the way for novel research directions and innovative ideas.

**FIGURE 1:**
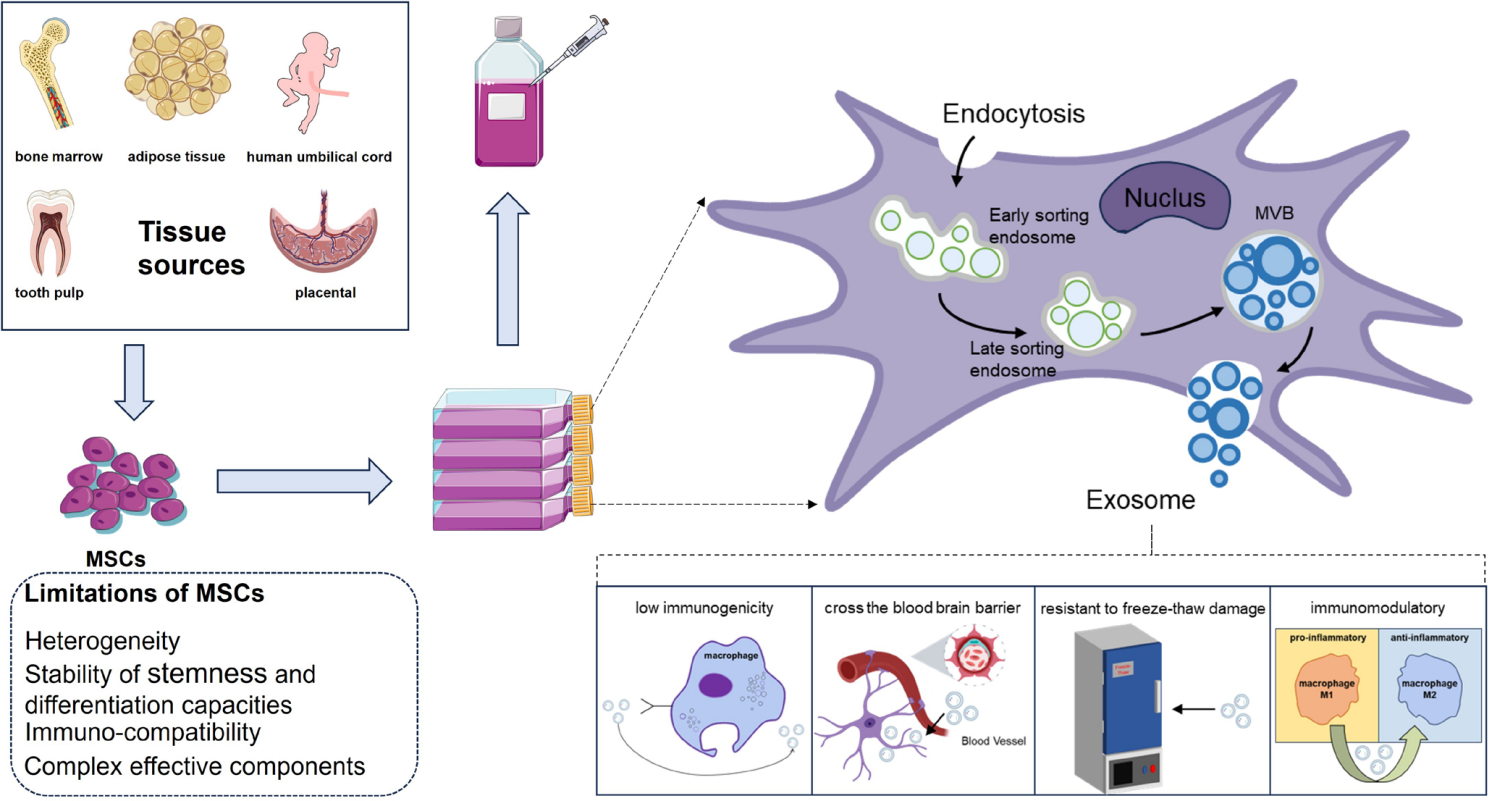
The production process of MSC‐Exos and characteristics of MSCs and MSC‐Exos. MSC‐Exos formation involves a series of steps including the endocytosis that leads to the generation of early sorting endosomes, the maturation into late sorting endosomes, the formation of multivesicular bodies, and the eventual release of exosomes. Limitations of MSCs included heterogeneity, stability of stem cell pluripotency and differentiation capacities, immunocompatibility, and complex effective components compared to MSCs, Four major advantages of MSC‐Exos are low immunogenicity, ability to cross the blood–brain barrier, resistance to freeze–thaw damage, and immunomodulatory effects, which can make exosomes promising candidates for therapeutic interventions in various diseases.

When compared to MSCs, MSC‐Exos exhibit several advantages in clinical applications (Figure 1). Notably, they possess superior resilience to freeze–thaw injury and maintain high stability in vivo [16, 17]. Additionally, their non‐replicative nature eliminates the tumorigenic risk associated with MSCs [16, 18]. Moreover, their low immunogenicity and ability to traverse the blood–brain barrier position them as formidable candidates in anti‐aging therapeutics [19, 20]. Therefore, this paper aims to provide a comprehensive review of the application of MSC‐Exos in age‐related diseases, delving into their mechanism of action, therapeutic effects, and future research directions. By thoroughly analysing and summarising existing studies, we aspire to offer valuable references and insights that can guide future research and foster the application and advancement of MSC‐Exos in the field of anti‐aging.

## 2 Biogenesis, Mechanism of Action, and Tissue Origin of Mesenchymal Stem Cell‐Derived Exosomes

Exosome biogenesis is a multistage cellular process that encompasses the formation of early endosomes to the release of mature exosomes (Figure 1). The exosome biogenesis begins with the cell membrane invagination, forming early sorting endosomes (ESE). These ESE mature into late‐sorting endosomes (LSE), undergoing morphological changes that include the formation of intraluminal vesicles (ILVs), which contain biomolecules from the parent cell. ILV formation is tightly regulated, particularly by the ESCRT complex. LSE then becomes a multivesicular body (MVB), encapsulating ILVs, and finally fuses with the cell membrane to release ILVs as exosomes into the extracellular space [21, 22, 23, 24]. During this biogenesis process, the main biomarkers inherited by exosomes include the tetraspanin family proteins such as CD63, CD81 and CD9, which are often used as consistent exosome markers and play crucial roles in facilitating the sorting of protein cargoes into exosomes [24]. Additionally, exosomes are known to contain heat shock proteins (HSPs), such as HSP70 and HSP90, which are involved in protein folding and stabilisation [24]. During the preparation and isolation of exosomes, it is customary to rely on size determination and the recognition of biomarkers to distinguish them from two other types of extracellular vesicles, including microvesicles and apoptotic bodies. In contrast, Microvesicles, are larger than exosomes and derived from the plasma membrane's outward budding. They are rich in membrane‐associated proteins like integrins and selectins, which play roles in cell adhesion and signalling [25]. Apoptotic bodies, formed during cell death, are the biggest one and contain organelles and DNA fragments [26]. They are identified by phosphatidylserine on their surface, which Annexin V can bind to, and may also be marked by thrombospondin and the complement protein C3b [25, 27]. At present, methods including differential centrifugation, precipitation, flushing separation, ultrafiltration, antibody affinity capture, microfluidic separation and mass spectrometry (MS) can be used to effectively isolate and prepare exosomes [28]. Regarding the storage of MSC‐Exos, the current consensus is that they can be temporarily preserved at 4°C for short‐term durations (1 day to several weeks), while long‐term storage requirements (several months to years) are best met by cryopreservation at ultra‐low temperatures of −80°C [17]. Compared to MSCs, MSC‐Exos exhibit remarkable resistance to freeze–thaw cycles. This enhanced stability throughout the freeze–thaw process renders MSC‐Exos more suitable for clinical applications and storage [16].

**FIGURE 2:**
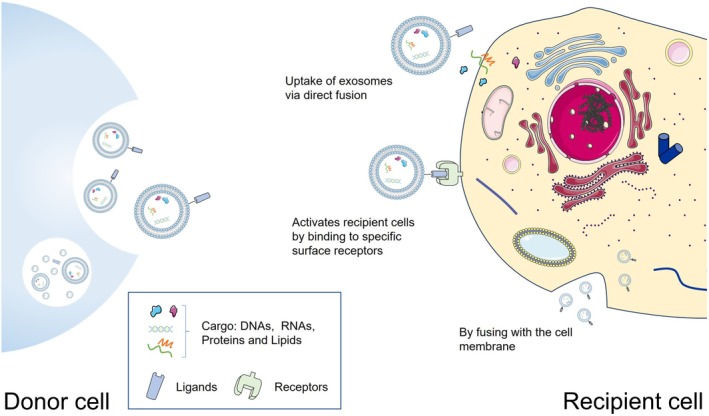
Mechanisms of Exosome‐Mediated Target Cell Activation and Cargo Transfer. Exosomes have three main mechanisms by which they interact with target cells. To begin with, intracellular signalling molecules released by exosomes can directly interact with target cells, thereby modulating their biological functions accordingly. Additionally, exosomes bind to specific surface receptors on the recipient cell surface, leading to the activation of the target cells. This activation can initiate a cascade of intracellular biological responses through signal transduction pathways. Finally, exosomes can be internalised by recipient cells via direct membrane fusion.

**FIGURE 3:**
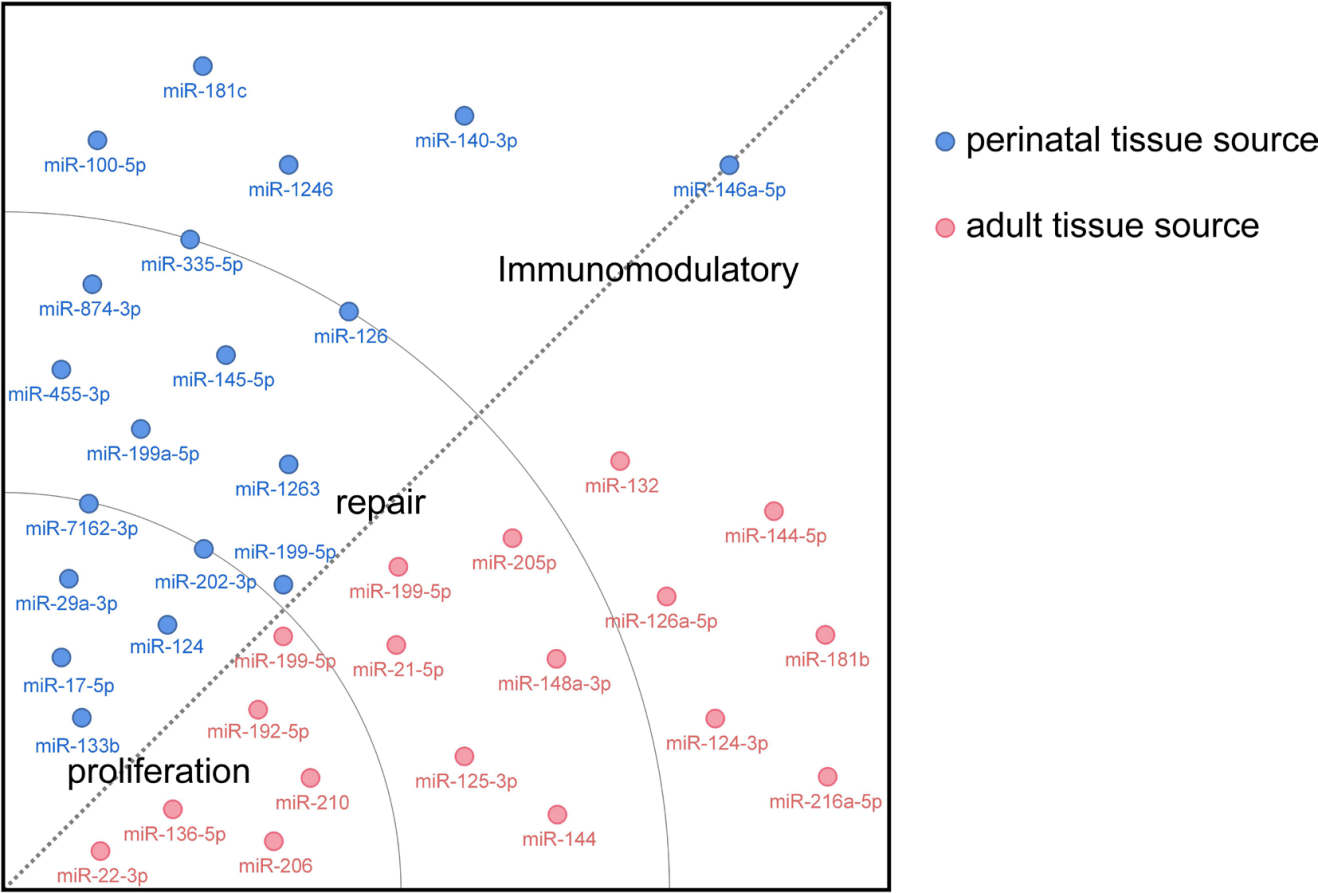
Classify the miRNA cargos in MSC‐Exos derived from different donors. Perinatal tissues, including the placenta, umbilical cord blood, and amniotic fluid, as well as adult tissues such as bone marrow and adipose tissue, are common sources of MSC‐Exos. MSC‐Exos derived from adult tissue sources contain miRNAs that exhibit superior regenerative and immunomodulatory capabilities, with perinatal tissue‐derived MSC‐Exos showing particular prominence in tissue repair and cellular regeneration.

As key mediators of intercellular communication, exosomes employ diverse and intricate mechanisms of action. These nanoscale vesicles can facilitate information transfer via direct binding of ligands on their membrane surface to receptors on target cells, or through membrane fusion with the target cell membrane. Additionally, exosomes release intracellular signalling molecules that bind directly to target cells, modulating their biological functions accordingly (Figure 2). Table 1 and Table 2 provide a comprehensive overview of the various mechanisms of action exhibited by MSC‐Exos derived from perinatal tissue and adult tissue sources, respectively. The nucleic acids, particularly microRNAs (miRNA) and long non‐coding RNAs (lncRNA), as well as proteins like 14–3‐3ζ proteins, that are harboured within these exosomes, play pivotal roles in disease therapeutics. Among these, miRNAs stand out as particularly significant players, as they are encapsulated within exosomes and function as molecular messengers, transmitting genetic information to recipient cells via cell‐to‐cell transfer, ultimately influencing gene expression and biological functions of the target cells. A range of studies has illuminated the crucial role of miRNAs in MSC‐Exos, particularly in facilitating intercellular communication and regulating biological functions. For example, the hUCMSC‐Exos derived miR‐335‐5p was found to mitigate inflammation in HK‐2 cells by downregulating TGF‐β1‐induced ADAM19 protein levels [29]. Additionally, miR‐133b derived from hUCMSC‐Exos augmented proliferation, cell cycle progression, migration, and invasion, while inhibiting apoptosis in HTR8‐S/Vneo and HPT‐8 cells [30]. pTEN, an oncogene, encodes proteins with phosphodiesterase activity that normally inhibit tumour cell growth and invasion. However, during wound healing, moderate levels of cell proliferation and angiogenesis are essential [31]. Consequently, modulating PTEN activity can promote these processes. Recent investigations have revealed that miR‐29a‐3p, delivered via hUCMSC‐Exos, promotes tendon healing through the PTEN/mTOR/TGF‐β‐1 signalling cascade [32]. Furthermore, miR‐125a‐3p derived from ADSC‐Exos has been shown to expedite wound healing and angiogenesis in mice by inhibiting PTEN in wound granulation tissues, presenting a novel therapeutic approach [33]. These findings underscore the therapeutic potential of MSC‐Exos and their miRNA cargo in regenerative medicine and wound healing. We have categorised and quantified the miRNAs exerting effects within exosomes derived from various tissues (Figure 3). Our analysis reveals that exosomes from perinatal tissues, including placenta, umbilical cord blood, and amniotic fluid, demonstrate superior regenerative capabilities and tissue repair properties. In contrast, MSC‐Exos derived from adult tissue sources show a more pronounced effect in both regeneration and immune regulation. Furthermore, the same miRNA can be expressed in MSC‐Exos derived from various sources, yet its functions may be contextually distinct. For instance, miR‐199‐5p is present in both BMSC‐Exos and hUMSC‐Exos. BMSC‐Exos are enriched with miR‐199a‐5p, which has been shown to promote the proliferation of neural stem cells [34]. In addition, miR‐199‐5p in hUMSC‐Exos ameliorates lung injury induced by mustard gas by activating the NRF2 signalling pathway, thereby reducing oxidative stress and inhibiting apoptosis.

**TABLE 1:** Overview of the various mechanisms of action of perinatal tissue‐derived MSC‐Exos.

Component	Mechanism	Instances
Human umbilical cord		
lncRNA MALAT1 [35]	lncRNA TUBB6/Nrf2 pathway	Inhibits inflammation and iron death
miR‐874‐3p [36]	Regulation of necrotic apoptosis	Reduces renal tubular epithelial cell damage
miR‐1246 [37]	Targeting PRSS23 and inhibiting the activation of the Snail/alpha‐smooth muscle Actin signalling	Attenuated hypoxia‐induced myocardial tissue damage
miR‐133b [30]	Targeting SGK1	Promoted proliferation, cell cycle progression, migration and invasion, and apoptosis in HTR8‐S/Vneo and HPT‐8 cells.
miR‐451a [38]	Targeting ADAM10	Limiting epithelial‐mesenchymal transition in hepatocellular carcinoma cells
miR‐181c [39]	Downregulation of the TLR4 signalling pathway	Reduction of burn‐induced inflammation
miR‐146a‐5p [40]	IRAK1/TRAF6 pathway	Reduction of microglia‐mediated neuroinflammatory responses and neurological deficits
lncRNA HCP5 [41]	Promotes ESR1 expression by binding to MSI2	Promotes the proliferation of ovarian granulosa cells
miR‐17‐5p [42]	Mitigates ROS accumulation and inhibits downstream target mRNA SIRT7	Promote ovarian cell proliferation
miR‐140‐3p [43]	miR‐140‐3p/FOXP1/Smad pathway	Anti‐fibrotic effects in human endothelial cells
miR‐7162‐3p [44]	Targeting 3′‐UTR in endometrial stromal cells (ESC) to regulate APOL6 expression	Protection of ESCs from mifepristone‐induced apoptosis and repair of damaged ESCs
miR‐140‐3p [45]	Inhibition of SGK1 upregulation	Alleviating rheumatoid arthritis
lnc‐CDHR [46]	Competitive binding of miR‐3149 inhibits target PTEN genes via the AKT/FOXO pathway	Mitigation of epithelial‐mesenchymal transition in the human peritoneal mesothelial cell line
miR‐100‐5p [47]	FZD5/Wnt/β‐catenin pathway	Inhibition of cellular progression and inflammatory response in eosinophils, thereby mitigating the progression of atherosclerosis
lncRNA UCA1 [48]	miR143/Bcl‐2/Beclin‐1 pathway	Against hypoxia/reoxygenation injury
miR‐199a‐5p [49]	CAV1/NRF2 pathway	Attenuates sulfur mustard‐associated oxidative stress
miR‐29a‐3p [32]	PTEN/mTOR/TGF‐β‐1 pathway	Tendon regeneration
miR‐202‐3p [50]	Regulate extracellular matrix remodelling	Promote endometrial repair
miR‐24‐3p [51]	Targeting Keap‐1 signalling	Hepatoprotective by inhibiting lipid accumulation, ROS generation, and inflammation
miR‐335‐5p [29]	Reduction of TGF‐β1‐induced ADAM19 protein levels	Reducing inflammation in HK‐2 cells
[52]	Regulation of m6A modification of ITG β4	Alleviated Lipopolysaccharide‐induced acute lung injury
miR‐126 [53]	Downregulation of HMGB1 signalling pathway	Reducing hyperglycemia‐induced retinal inflammation
lncRNA MALAT1 [54]	Inhibition of the NF‐kappa B/TNF‐α signalling pathway	Delaying the progression of aging and age‐related diseases
miR‐455‐3p [55]	Targeting PI3K signalling	Inhibition of macrophage activation and cytokine production by lipopolysaccharide attack for acute inflammatory liver injury.
miR‐100‐5p [56]	Elimination of oxidative free radicals through its target NOX4	Treatment of articular chondrocyte damage and osteoarthritis
miR‐21 [57]	Down‐regulates LATS1, thereby reducing phosphorylated LOXL2 and YAP	Promote oestrogen secretion from ovarian granulosa cells
miR‐27b‐3p [58]	Mediates YAP downregulation to inhibit LOXL2 expression	Alleviating liver fibrosis
miR‐223 [59]	Reduced donor T‐cell migration	Alleviates acute graft‐versus‐host disease
miR‐124 [60]	Down‐regulation of the target Foxg1	Liver regeneration after partial hepatectomy
lncRNA PTENP1 [61]	Competitive binding of miR‐10a‐5p to stabilise PTEN	Inhibit the glioma cell progression
miR‐145‐5p [62]	Attenuates oxidative damage and apoptosis in granulosa cells	Reducing ovarian damage
lncRNA GAS5 [63]	Competitive binding to miR‐21 regulates lncRNA GAS5/miR‐21/PTEN through the Wnt/β‐catenin signalling pathway	Mitigation of high glucose‐induced epithelial mesothelial transformation in human peritoneal mesothelial cells
miR‐1263 [64]	Targeting Mob1 in recipient cells, thereby activating YAP expression	Reduction of BMSC apoptosis in Disuse osteoporosis
14–3‐3ζ protein [65, 66, 67]	Upregulate SIRT1 expression levels in HaCaT cells by trafficking 14–3‐3ζ protein to relieve UV radiation‐ and H_2_O_2_‐induced oxidative stress damage	Reduces UV radiation‐induced ROS production and DNA damage, promotes the activation of autophagy, and exerts cytoprotective effects
	Increased autophagy levels	Reduction of cisplatin‐induced nephrotoxicity
	Up‐regulation of autophagy level in HK‐2 cells	Reduction of cisplatin‐induced nephrotoxicity
TSG‐6 [68]	A secreted protein with anti‐inflammatory and tissue‐protective properties	Protecting the intestinal barrier and modulating the immune response
GPX1 [69]	Up‐regulation of ERK1/2 and Bcl‐2 and down‐regulation of IKKB/NFkB/casp‐9/−3 pathway	reduction of hepatic ROS and inhibition of oxidative stress‐induced apoptosis
Human deciduous exfoliated		
miR‐100‐5p miR‐1246 [70]	Downregulates VEGF expression	Inhibition of angiogenesis
Human amniotic fluid		
miR‐146a‐5p miR‐548e‐5p [71]	Regulation of nuclear factor κB, AKT, and mitogen‐activated protein kinase protein phosphorylation	Anti‐inflammatory

**TABLE 2:** Overview of the various mechanisms of action of adult‐derived MSC‐Exos.

Component	Mechanism	Instances
Adipose tissue		
miR‐125a‐3p [33]	Inhibition of PTEN in mouse wound granulation tissue	Angiogenesis, wound healing
miR‐29a [72]	Inhibition of the TGF‐β2/Smad3 signalling pathway	Inhibits scar formation
lncRNA IFNG‐AS1 [73]	Acts as a molecular sponge through the miR‐21a‐3p/PI3K/AKT signalling pathway	Neurogenesis
miR‐216a‐5p [74]	Regulation of the HMGB1/TLR4/NF‐kappa B signalling pathway	Induces macrophage M2 polarisation and attenuates colitis.
miR‐148a‐3p [75]	Preventing PTEN and triggering PI3K/Akt signalling	Angiogenesis, wound healing
EGR‐1 [76]	Activation of the lncRNA‐SENCR/VEGF‐A axis	Angiogenesis, wound healing
VEGF MCP‐2/4 [77]	Promotion of VEGF‐R expression	Angiogenesis
Bone marrow		
miR‐146a [78]	Targeting IRAK1, TRAF6 and IRF5 in immune cells	Anti‐inflammatory
miR‐144‐5p [79]	Mediated PTEN inhibition leads to the activation of PI3K/AKT signalling	Induction of apoptosis or cell cycle arrest in the G1 phase
lncRNA H19 [80]	Competitive binding of miR‐467 to affect Hoxa10 expression	Affects osteoblast differentiation
miR‐30b‐5p [81]	EZH2/PI3K/AKT axis	Promoting apoptosis and arresting tumourigenesis in non‐small cell lung cancer cells
lncRNA TUC339 [82]	Increased expression of TUC339	Promoting macrophage M1 to M2 polarisation, inhibiting inflammation, and promoting chondrocyte activity to ameliorate osteoarthritis
circHIPK3 [83]	Acts as a miR‐29a‐5p sponge and plays a role in mitochondrial autophagy by targeting miR‐29a‐5p and PINK1	Promoting osteoblastic differentiation of MC3T3‐E1 cells
miR‐181b [84]	Regulation of the interleukin 10/STAT3 pathway	Suppression of neuroinflammation after traumatic brain injury
miR‐199a‐5p [34]	GSK‐3 β/β‐catenin signalling.	Promote neural stem cell proliferation
miR‐202‐5p [85]	Targeting CMPK2	Inhibition of up‐regulation of focal death proteins, alleviate lung ischemic‐reperfusion injury
miR‐21‐5p [86]	Inhibition of PDCD4 expression	Reduction of β‐cell apoptosis in the early stages of islet transplantation
miR‐144 [87]	Targeting the PTEN/AKT pathway	Inhibition of apoptotic injury under hypoxic conditions
miR‐136‐5p [88]	Targets Elf3 and can downregulate its expression	Promotes chondrocyte migration with increased expression of collagen II, aggregated glycans, and SOX9 and decreased expression of MMP‐13.
miR‐96 [89]	Inhibition of the Rac/nuclear factor‐κ B signalling pathway	Protection of myocardium from doxorubicin‐induced toxicity
miR‐206 [90]	Decrease Elf3	Promote proliferation and differentiation of osteoblasts in OA
miR‐124‐3p [91]	Inhibiting Ern1 and promoting M2 polarisation	Improvement of spinal cord ischemia–reperfusion and its associated injuries
lncRNA KLF3‐AS1 [92]	miR‐206/ubiquitin‐specific peptidase 22‐axis stabilisation sirtuin‐1	Improved neurological function in mice with middle cerebral artery occlusion promoted cell viability inhibited apoptosis and inflammatory injury, and reactive oxygen species production
Tooth pulp		
lncRNA Ankrd26 [93]	Regulates miR‐150‐TLR4 signalling	Promote endodontic restoration

## 3 Mechanisms of Anti‐Aging in Mesenchymal Stem Cell‐Derived Exosomes

**FIGURE 4:**
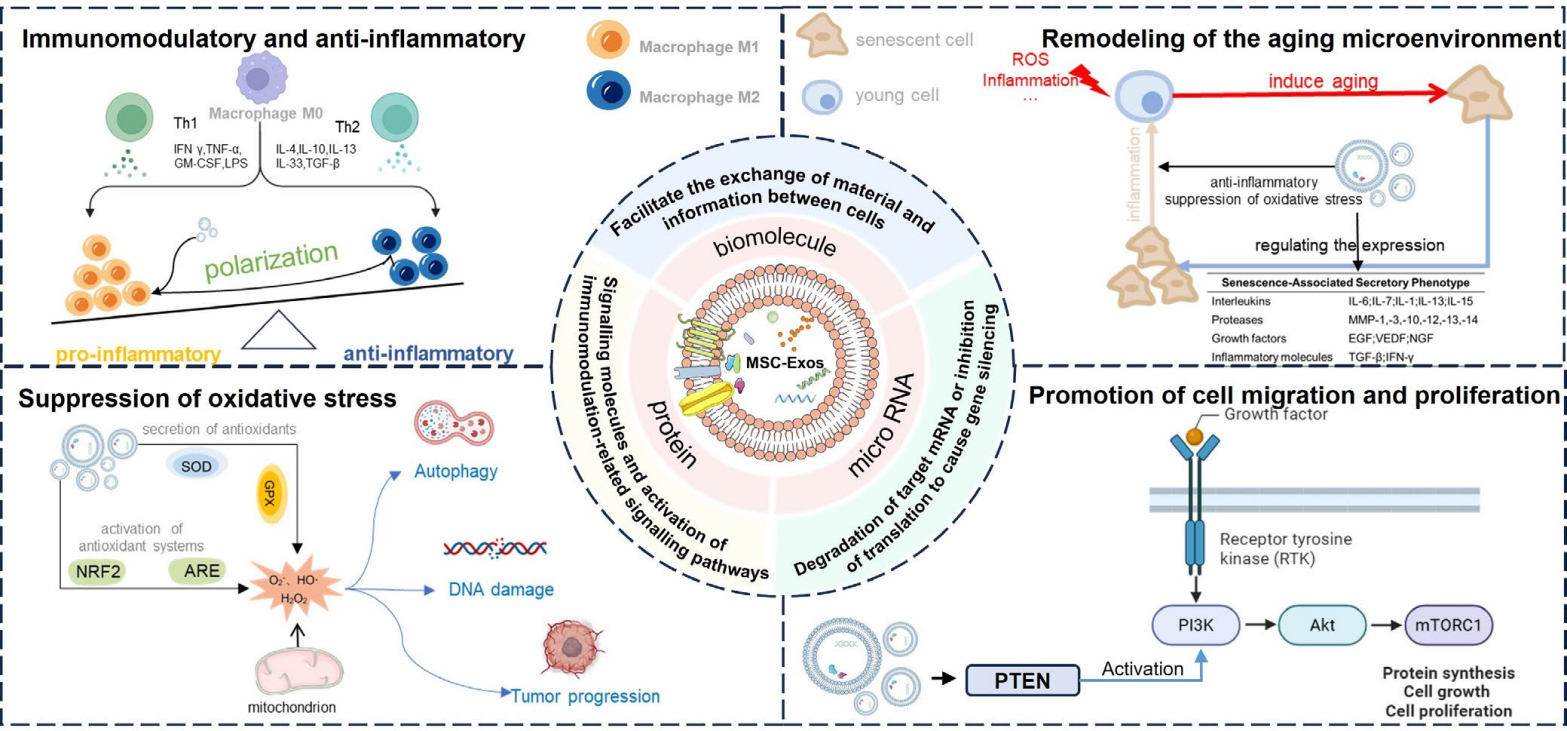
Mechanisms of MSC‐Exos in the treatment of age‐related diseases. A Immunomodulatory and anti‐inflammatory, MSC‐Exos mediate immunomodulatory and anti‐inflammatory effects by regulating the polarisation of T cells and macrophages, promoting an anti‐inflammatory state while inhibiting pro‐inflammatory responses; B Suppression of oxidative stress, MSC‐Exos mitigate oxidative stress by activating cellular antioxidant defence mechanisms, including NRF2/ARE signalling pathway. Through the upregulation of antioxidant enzymes (e.g., SOD, GPx), MSC‐Exos facilitate the reduction of ROS and prevent autophagy oxidative DNA damage, thereby inhibiting pathways associated with tumour progression and cellular senescence; C Promotion of cell migration and proliferation, MSC‐Exos activate the PI3K/AKt/mTOR signalling pathways by PTEN to promotion protein synthesis, cell growth, and cell proliferation;D Remodelling of the aging microenvironment, MSC‐Exos regulate the aging microenvironment by modulating the expression of inflammatory and anti‐inflammatory factors within the SASP. Through the suppression of pro‐inflammatory cytokines and growth factors, MSC‐Exos alleviate age‐related inflammation and mitigate cellular aging processes that are exacerbated under stress conditions.

MSC‐Exos mitigate age‐related diseases across multiple systems through diverse signalling pathways. The specific mechanisms of action of exosomes include immunomodulatory and anti‐inflammatory effects, suppression of oxidative stress, promotion of cell migration and proliferation, and remodelling of the aging microenvironment (Figure 4).

### 3.1 Immunomodulatory and Anti‐Inflammatory

Inflammatory aging denotes a state of persistent, low‐grade inflammation accompanying the aging process and shows a propensity to escalate with advancing age [94]. A cardinal feature of inflammatory aging is the perturbation in the equilibrium between pro‐inflammatory and anti‐inflammatory mediators. Molecularly, cellular senescence precipitates the secretion of pro‐inflammatory factors collectively referred to as senescence‐associated secretory phenotype (SASP). These SASPs encompass a spectrum of inflammatory mediators, including cytokines, chemokines and growth factors, which are implicated in the perpetuation of chronic inflammation and the progression of cellular senescence [95]. At the cellular level, immune cells, pivotal in the regulation and clearance of senescent cells, undergo functional decline with age. This senescence of the immune system is implicated in the compromised clearance of senescent cells and the accumulation of pro‐inflammatory factors, thereby contributing to heightened levels of inflammation. This evolving inflammatory milieu is posited to further the aging process in a cyclical and self‐sustaining manner [95]. If unaddressed, the relentless accrual of inflammatory processes at the molecular and cellular strata can precipitate pathological aging of organs. Persistent inflammation impedes reparative mechanisms and escalates the risk of geriatric disease [96].

MSC‐Exos have risen as influential regulators of immune cell function, integral to the maintenance of immune system homeostasis and the attenuation of age‐associated inflammatory responses. Enriched with inflammatory modulators, including transforming growth factor‐beta (TGF‐β), nitric oxide (NO), prostaglandin E2 (PGE2), and interleukin‐10 (IL‐10), MSC‐Exos exert anti‐inflammatory effects. They operate by curbing the release of inflammatory mediators and by orchestrating immune responses. Specifically, TGF‐β inhibits the activity of a wide range of immunoreactive cells while inducing an immunosuppressive cell phenotype to maintain immune homeostasis [97]. NO, a vascular modulator, dilates blood vessels to increase blood flow to inflamed areas, facilitating nutrient delivery and waste clearance, while also suppressing inflammatory cell activity to reduce inflammation. Additionally, MSC‐Exos can influence lymphocyte differentiation [98]. Research has discovered that MSC‐Exos are capable of inhibiting the secretion of IL‐1β and TNF‐α while promoting the secretion of TGF‐β. Further studies have indicated that MSC‐Exos can induce the transformation of Th1 cells towards Th2 cells [99]. Concurrently, MSC‐Exos are enriched with negative feedback immunomodulatory molecules, such as Programmed Death‐Ligand 1 (PD‐L1) and TGF‐β. PD‐L1 is instrumental in dampening T‐cell immune responses and fostering the expansion of regulatory Tregs [100], while TGF‐β facilitates the differentiation of monocytes into Tregs [101]. In the experimental study on immunomodulatory effects on experimental type‐1 autoimmune diabetes (T1DM), ADSC‐Exos exert therapeutic effects on autoimmune T1DM by expanding the population of Tregs and their products. Notably, there is no change observed in the proliferation index of lymphocytes, which suggests that these exosomes could offer a more effective and practical therapeutic option [102]. MSC‐Exos have demonstrated the capacity to regulate macrophage polarisation. In a study conducted by Zhao, ADSC‐Exos were observed to act upon macrophages, inducing high levels of M2‐associated arginase‐1 (Arg‐1) and IL‐10 [103]. This action inhibits the inflammatory responses of macrophages stimulated by lipopolysaccharide (LPS) in conjunction with IFN‐γ. The ADSC‐Exos, which induced an Arg‐1^high^M2 phenotype, exhibited elevated levels of phosphorylated signal transducer and activator of transcription 3 (p‐STAT3). This finding suggests that ADSC‐Exos may be a significant source of p‐STAT3 for recipient macrophages. ADSC‐Exos that lacked STAT3 activation failed to induce Arg‐1 expression in macrophages, whereas the knockdown of STAT3 in macrophages did not affect the Arg‐1 expression induced by ADSC‐Exos. This underscores the role of exosome‐borne active STAT3 in the transcriptional activation of Arg‐1, leading to the induction of an anti‐inflammatory M2 macrophage phenotype [103]. Another study has demonstrated that MSC‐Exos modulate immune cell responses by activating M2‐type macrophages, inhibiting M1‐type macrophages, and enhancing the expression of anti‐inflammatory cytokines IL‐10 and TGF‐β, thereby promoting tissue repair. Contrary to lipopolysaccharide, they induce high levels of anti‐inflammatory IL10 and TGFβ1 transcripts at 3 and 72 h, while the levels of pro‐inflammatory IL1B, IL6, TNFA, and IL12P40 transcripts are significantly reduced within 3 h [104].

### 3.2 Suppression of Oxidative Stress

Oxidative stress, a critical catalyst in the aging process, arises from an imbalance between the production of reactive oxygen species (ROS) and a biological system's capacity for detoxification. This imbalance is particularly pronounced as organisms age, often precipitating mitochondrial dysfunction that escalates ROS levels, thereby further impairing mitochondrial function and cellular energy metabolism. The consequent oxidative stress can induce DNA damage, which, if persistently unrepaired, may lead to genomic instability—a significant contributor to the aging phenotype. Moreover, oxidative stress accelerates the shortening of telomeres, triggering cellular senescence as cells exhaust their capacity for division [105]. The peroxidation of lipids in cellular membranes by ROS disrupts membrane fluidity and function, propelling cellular aging [106]. Additionally, oxidative stress activates inflammatory pathways, fostering chronic inflammation that is both a hallmark of aging and a risk factor for a variety of age‐related diseases. Proteins are not spared by oxidative stress, which can induce modifications leading to misfolding and aggregation, especially in neurodegenerative conditions [107]. Furthermore, oxidative stress can precipitate a decline in immune function, reducing the body's ability to fight infections and increasing the risk of autoimmune and neoplastic diseases.

MSC‐Exos are recognised for their potential to mitigate the oxidative stress inherent to the aging process through a variety of mechanisms. MSC‐Exos have emerged as pivotal players in neutralising oxidative stress by scavenging ROS, thereby preventing the cytoarchitectural damage and dysfunction that ensue [108]. These exosomes are laden with antioxidants such as glutathione peroxidase (GPX) and superoxide dismutase (SOD), which are instrumental in bolstering the antioxidant defences of recipient cells. Notably, GPX1, secreted by hUCMSC‐Exos, has been shown to degrade oxidative stress‐inducing agents such as carbon tetrachloride (CCl_4_) and hydrogen peroxide (H_2_O_2_) [69]. Additionally, ADSC‐Exos have been reported to reduce high glucose‐induced oxidative stress by increasing the activity of SOD2, thus alleviating mitochondrial dysfunction [109]. The inclusion of Vitamin E in MSC‐Exos further amplifies their antioxidant capabilities by directly neutralising free radicals and reducing lipid peroxidation, safeguarding cell membranes from oxidative damage [110]. Beyond direct ROS neutralisation, bioactive molecules within exosomes activate the intracellular antioxidant system, modulating the expression of genes that constitute the endogenous antioxidant defence system. This activation enhances the cells' overall antioxidant capacity, providing a comprehensive defence against oxidative stress. The Nrf2/ARE signalling pathway is integral to the cellular response to oxidative stress. The transcription factor NF‐E2‐related factor 2 (Nrf2) binds to the antioxidant‐responsive element (ARE), a cis‐acting element that mediates the transcriptional activation of protective genes. Activation of this pathway serves to safeguard cells from oxidative stress‐induced apoptosis [111]. ADSC‐Exos have been shown to activate this pathway, initiating the transcription and translation of antioxidant genes, as well as the expression of antioxidant enzymes and related proteins. This activation helps to reduce intracellular ROS levels and ameliorate mitochondrial and cellular damage [112, 113]. The miRNAs carried by MSC‐Exos, such as the miR‐17‐92 cluster, counteract oxidative stress by targeting the downregulation of PTEN, which in turn activates the PI3K/Akt/mTOR/GSK‐3β signalling pathway. This activation may contribute to the upregulation of antioxidant pathways within the cell, further enhancing the cell's defence mechanisms against oxidative stress [114].

Autophagy and oxidative stress are interconnected physiological processes that play pivotal roles in cellular homeostasis and disease progression. During autophagy, damaged organelles and proteins are sequestered into autophagosomes and then transported to lysosomes for degradation, thereby regulating intracellular material recycling and maintaining cellular redox balance. For instance, studies have shown that under conditions of nutrient deprivation, autophagy reduces ROS production by degrading impaired mitochondria [115]. MSC‐Exos may activate mitochondrial autophagy, a process that selectively removes damaged mitochondria, thereby helping to reduce intracellular ROS levels. The PI3K/Akt/mTOR pathway is a significant regulatory axis of autophagy, with MSC‐Exos stimulating autophagy in recipient cells by transferring bioactive molecules, such as Beclin‐1 and LC3, to eliminate intracellular senescence‐associated proteins and organelles [116]. Another study has demonstrated that MSC‐Exos enhance mitochondrial autophagy by upregulating FOXO3a expression, thereby protecting microglia from ischemia/reperfusion (I/R)‐induced pyroptosis and attenuating subsequent neuronal damage [117].

### 3.3 Promotion of Cell Migration and Proliferation

Aging is characterised by a complex series of biological changes within cells, leading to a gradual decline in their functional capabilities and ultimately resulting in cellular senescence and death. The voids left in tissues and organs necessitate timely replenishment to preserve the homeostatic functioning of the organism. In this context, cell proliferation and migration emerge as particularly critical processes. Cell proliferation forms the basis for the generation of new cells to replace those that have become senescent or have died. This process is essential for maintaining tissue integrity and repairing damaged structures. The fidelity of cell proliferation hinges on a delicate balance of biochemical reactions, which can be compromised by the accumulation of genetic damage and the decline in organelle function—hallmarks of aging [118, 119]. Parallel to cell proliferation, cell migration is equally vital for orchestrating immune responses and facilitating tissue regeneration. Cell migration not only shields the organism from infections and diseases but also fosters tissue repair and renewal. The ability of cells to migrate is influenced by various factors, including cytoskeletal remodelling, alterations in adhesion molecule expression, and changes in the extracellular matrix composition. MSC‐Exos may play a role in combating aging by promoting cell proliferation and migration through the activation of signalling pathways associated with these processes.

MSC‐Exos are enriched with a plethora of bioactive components, including growth factors, cytokines, miRNAs, and other biologically active molecules. Growth factors such as vascular endothelial growth factor (VEGF), epidermal growth factor (EGF), and platelet‐derived growth factor (PDGF) within MSC‐Exos play pivotal roles in promoting cell proliferation, differentiation and angiogenesis, which are essential for tissue repair [120]. Cytokines like HGF and IGF contribute to cell proliferation and differentiation, and are involved in the synthesis of the extracellular matrix. MiRNAs, including miR‐125a, miR‐21, and miR‐146a, facilitate angiogenesis in endothelial cells and modulate gene expression to enhance lymphocyte proliferation and differentiation [121]. Collectively, these components exert their effects through various signalling pathways and mechanisms, which modulate cell signalling pathways and regulate cell proliferation and apoptosis. For instance, they upregulate anti‐apoptotic genes like Bcl‐xL, downregulate pro‐apoptotic genes like Casp1, and activate extracellular signal‐regulated kinases to promote cell proliferation and anti‐apoptosis, essential for tissue repair [122]. Thereby, MSC‐Exos can promote cell proliferation and migration, which are crucial in the therapeutic management of age‐related diseases. Research indicates that transcription factors in MSC‐Exos are crucial for the maintenance of MSC pluripotency and self‐renewal by promoting the expression of pluripotency‐associated genes and inhibiting those related to differentiation [123]. Additionally, studies have shown that under hypoxic conditions, miR‐155 in MSC‐Exos positively modulates the expression of immunomodulatory factors in MSCs, thus preserving their regenerative capacity. Furthermore, exosomes enhance the proliferation and migration of target cells, thereby exerting anti‐aging effects in conditions such as osteoarthritis. Activation of this pathway is typically initiated by extracellular stimuli. miR‐21, a miRNA involved in numerous biological processes, including cell proliferation and migration, can activate the PI3K/Akt pathway, which is integral to regulating cell metabolism, proliferation, survival, growth, and angiogenesis, by targeting genes like the tumour suppressor gene pTEN [124]. This activation enhances the proliferation and migration of skin fibroblasts, contributing to wound healing and skin regeneration [125]. Activation of this pathway is typically initiated by extracellular stimuli, such as growth factors, cytokines, or hormones. miR‐21, a miRNA involved in numerous biological processes, including cell proliferation and migration, can activate the PI3K/Akt pathway by targeting genes like the tumour suppressor gene pTEN [126, 127]. MSC‐Exos contribute to the rejuvenation of cells by inducing autophagy, promoting DNA repair, and facilitating angiogenesis. MSCs also harbour DNA repair enzymes, including PARP1 and APE1, capable of repairing DNA damage in recipient cells, thus slowing the aging process [124]. Furthermore, MSCs stimulate vascular neogenesis, improve tissue perfusion, and decelerate cellular senescence by secreting growth factors like VEGF, EGF and PDGF.

### 3.4 Remodelling of the Aging Microenvironment

The aging microenvironment refers to the local milieu surrounding target cells, encompassing elements such as the extracellular matrix (ECM), cytokines, and neighbouring cells, which collectively influence cellular behaviour and function [128]. Given that the SASP is a result of aged cells expressing and secreting a variety of extracellular modulators, including the pro‐inflammatory factors mentioned earlier that contribute to inflamm‐aging, the generated ROS can further stimulate cellular oxidative stress and the onset of senescence. Additionally, factors such as matrix remodelling enzymes affect the modification of the ECM, the fate of neighbouring cells, and intercellular communication, thereby coordinating the modification of the microenvironment [129]. The aging microenvironment, as proposed by the inflamm‐aging hypothesis, is characterised by a deteriorating milieu influenced by the SASPs, which are secreted via paracrine mechanisms. These factors not only initiate chronic inflammation but also induce senescence in neighbouring normal cells, establishing a self‐perpetuating cycle [130]. These factors dynamically, interactively, and adaptively influence the microenvironment, collectively constituting the aging microenvironment. Consequently, researchers have explored the reshaping of the senescent microenvironment as a means to mitigate the effects of aging. The stem cell microenvironment also declines with age, with a noticeable decrease in stem cell numbers and functionality, thus limiting the regenerative capacity of tissues. Aging induces alterations in metabolic pathways, affecting cellular energy production and metabolic waste clearance, leading to a progressive decline in cellular and tissue function. Furthermore, modifications in cellular signalling pathways with age influence how cells respond to growth factors, hormones, and other regulatory signals [131, 132], profoundly affecting cell proliferation, differentiation and survival.

MSC‐Exos play a crucial role in regulating the aging microenvironment. They participate in intercellular communication by carrying various bioactive molecules, influencing neighbouring cells, and exerting a regulatory effect on the aging microenvironment. The ability of MSC‐Exos to mitigate the effects of cellular senescence is particularly noteworthy [128]. They achieve this by modulating specific components of the SASP, which typically consists of pro‐inflammatory cytokines, chemokines, and ECM remodelling factors that can adversely affect neighbouring cells. By reducing the secretion of these detrimental factors, MSC‐Exos help restore homeostasis within the senescent microenvironment. Furthermore, MSC‐Exos influence critical cellular processes, including apoptosis, growth, proliferation, and differentiation. They achieve this by interacting with the ECM, which provides structural support and biochemical signals necessary for cell survival and function. This interaction allows MSC‐Exos to transmit bioactive molecules that alter the transcriptomic and proteomic profiles of recipient cells, thereby enhancing their regenerative potential and promoting cell survival [133]. The bioactive molecules carried by MSC‐Exos, such as growth factors and cytokines, can stimulate the proliferation and self‐renewal of stem cells, maintaining a stable and continuous supply of stem cell pools. Pro‐angiogenic factors in MSC‐Exos promote blood vessel neovascularization in the stem cell microenvironment, providing better blood supply and nutritional conditions. By regulating signalling pathways associated with cellular senescence, MSC‐Exos can slow the aging process of stem cells and maintain their functionality and regenerative capacity. The Hippo signalling pathway, an evolutionarily conserved pathway originally discovered in Drosophila and homologous in mammals, plays a key role in tissue regeneration, cell proliferation, and apoptosis [134, 135]. The pathway's core is a kinase cascade reaction involving multiple protein interactions. Mst1/2 kinase, bound to the Sav1 protein, activates Lats1/2 kinase, which phosphorylates and inhibits the transcriptional co‐activators YAP and TAZ. Phosphorylated YAP and TAZ are retained in the cytoplasm, unable to enter the nucleus. When the Hippo signalling pathway is inhibited, YAP and TAZ translocate to the nucleus, bind to the TEAD family of transcription factors, and activate genes that promote cell proliferation and inhibit apoptosis. The anti‐apoptotic effects of hUCMSC‐Exos on BMSCs in wasting osteoporosis involve the exosome miR‐1263, which inhibits the Mob1 inhibition of the BMSCs Hippo signalling pathway [64]. Moreover, MSC‐Exos provide a healthier living environment for stem cells by inhibiting oxidative stress and improving the inflammatory state of the stem cell microenvironment.

## 4 Clinical Application of Mesenchymal Stem Cell‐Derived Exosomes in Anti‐Aging

Biologists believe that aging may have an important internal relationship with many common diseases and that aging may increase the risk of a variety of diseases, including osteoarthritis (OA), skin aging, cardiovascular disease (CVD) and Alzheimer's disease (AD).

### 4.1 Mesenchymal Stem Cell Exosomes in Osteoarthritis

OA is the most common degenerative joint disease, with aging and obesity as major risk factors. Senescent cells, particularly in articular cartilage, inhibit stem cell differentiation, affecting joint regeneration [136]. Their removal can slow post‐traumatic OA progression, possibly due to reduced SASP. Specifically, in the context of OA, senescent p16INK4a‐positive cells have been demonstrated to induce the production of matrix metalloproteinases (MMPs) as part of the SASP. SASP, containing pro‐inflammatory cytokines and MMPs, degrades the ECM, contributing to OA's structural decline. Aging also brings epigenetic changes affecting chondrocyte function and stress response [137], mitochondrial dysfunction causing oxidative stress and energy deficits [138], and metabolic shifts leading to ECM component alterations, increasing cartilage wear [139]. Growth factor response modifications with age can impair chondrocyte maintenance of the cartilage matrix and response to signals. These factors—senescent cells, epigenetic alterations, mitochondrial issues, metabolic changes, and growth factor dysregulation—create a microenvironment that accelerates chondrocyte aging and joint degeneration.

MSC‐Exos have demonstrated remarkable potential in modulating cellular processes that are essential for maintaining cartilage health and facilitating its regeneration. In a study by Rilla et al., the intra‐articular injection of BMSC‐Exos into a rat model of osteoarthritis led to a significant enhancement in the content of type II collagen and proteoglycans within the cartilage tissues [140]. Concurrently, there was a notable decrease in the expression of matrix‐degrading enzymes such as MMP‐13 and aggrecanase, effectively promoting the repair of the cartilage matrix [140]. MSC‐Exos are equipped with a plethora of angiogenic factors, including VEGF, FGF, and PDGF. These factors are instrumental in stimulating neovascularization and augmenting blood supply to the affected areas [141]. The enhanced blood flow delivers vital oxygen and nutrients to the cells, underpinning their survival and reparative activities, while also aiding in the clearance of metabolic waste. This collective action fosters an auspicious environment conducive to chondrocyte survival. Several studies have attested to the capacity of MSCs to regulate cell proliferation and apoptosis, bolster extracellular matrix formation, and invigorate cartilage regeneration. Kuang et al. discovered that hUCMSC‐Exos could curb chondrocyte apoptosis by dampening the activities of B‐cell lymphoma‐2‐related promoter protein and cysteinyl aspartate‐specific protease‐3 through the Akt signalling pathway [142]. The Wnt signalling pathway, a cardinal signalling system in cell biology, governs cell proliferation, migration, and death. Its over‐activation has been implicated in apoptosis [143]. BMSC‐Exos, through the modulation of Wnt signalling, may attenuate chondrocyte apoptosis, thus mitigating the progression of osteoarthritis and other degenerative conditions [144]. Moreover, the Wnt pathway is intricately involved in the inflammatory response [145]. By tempering the Wnt signalling pathway, BMSC‐Exos may alleviate joint inflammation and ameliorate the symptoms of arthritis [144]. MSC‐Exos have been observed to counteract inflammatory senescence by diminishing the expression of inflammatory mediators, thereby indirectly hindering the onset of osteoarthritis. In vitro, studies have shown that the addition of MSC‐Exos to a chondrocyte culture medium, under stimulation with IL‐1β, resulted in the suppression of inflammatory factors such as TNF‐α and IL‐6 [146]. Vonk's research further elucidated that BMSC‐Exos impeded the phosphorylation of NF‐κB inhibitory proteins, culminating in the inhibition of the NF‐κB signalling cascade and a decrement in the expression of related inflammatory factors [147].

### 4.2 Mesenchymal Stem Cell‐Derived Exosomes in Skin Aging

The skin, as the body's largest tissue, shows aging effects from external exposure. Theories include oxidative stress, photoaging, and inflammation. ECM degradation, supported by increased MMPs and decreased inhibitors like TIMP‐1, leads to skin sagging and reduced elasticity [148]. UV radiation boosts MMPs, causing DNA damage and photoaging [149, 150]. Senescent cells, more common in older skin, release inflammatory SASP, which degrades ECM and accelerates aging. Fibroblast proliferation arrest and SASP increase also contribute to aging by impairing skin repair [151]. Age‐related skin microenvironment changes, such as reduced hydration and dermal thinning, weaken the skin's barrier and moisture retention [152]. Decreased lipids and glycosaminoglycans further disrupt skin structure and hydration.

A plethora of research has illuminated the capacity of exosomes MSC‐Exos to rejuvenate and regenerate damaged skin tissues through a myriad of mechanisms. Specifically, human umbilical cord‐derived mesenchymal stem cell exosomes hUCMSC‐Exos have demonstrated the ability to modulate the synthesis and degradation of the ECM, thereby mitigating the aging process of skin tissues. This is achieved by enhancing the production of collagen and elastin, key structural proteins that maintain skin integrity and elasticity, and by dampening the activity of MMPs, enzymes that can degrade these proteins when overactive, leading to a slowdown in the aging process [153]. HUCMSC‐Exos have been shown to stimulate the proliferation and migration of fibroblasts damaged by ultraviolet radiation. By optimising collagen deposition and stimulating elastin production, hUCMSC‐Exos expedite the healing of skin compromised by injury [141]. The exosomes are laden with growth factors that serve as potent activators of skin stem cells, thereby fostering the regeneration of damaged skin. These growth factors include PDGF, which synergize to augment the proliferation and differentiation of skin cells and hasten wound healing [154]. Moreover, hUCMSC‐Exos contain a constellation of bioactive molecules that can attenuate the detrimental effects of UV radiation [155]. Through the SIRT1 pathway, these exosomes can transport protective proteins such as 14–3‐3ζ to HaCaT cells, mediating a reduction in oxidative stress and inflammation, and effectively safeguarding skin cells from UV‐induced damage [65]. In summation, the collective findings underscore the proficiency of MSC‐Exos in facilitating the resuscitation of physiological functions in aging skin, offering a trove of opportunities for innovative treatments that could transform the landscape of dermatological care.

### 4.3 Mesenchymal Stem Cell‐Derived Exosomes in Cardiovascular Disease

Aging affects the heart and blood vessels, increasing susceptibility to CVD. In aged rats, cardiac angiotensin II levels rise, promoting vasoconstriction and hypertension, which are key in CVD development [156]. Mitochondrial dysfunction with age can lead to energy deficits, oxidative stress, and cellular damage within the heart. Inflammation, central to atherosclerosis, causes arterial plaque buildup, impeding blood flow and heightening the risk of heart attacks and strokes [157]. Anti‐inflammatory therapies have shown promise in CVD secondary prevention, emphasising inflammation's role in the disease. Cellular senescence in the heart, with the accumulation of non‐dividing, altered‐secretory senescent cardiomyocytes, is linked to CVD progression [158]. Their clearance post‐myocardial infarction improves outcomes, suggesting a therapeutic potential. Additional contributors to age‐related CVD include reduced vascular elasticity, endothelial dysfunction and altered lipid metabolism, leading to atherosclerotic plaque accumulation. Aging also impacts the heart's electrical system, potentially causing arrhythmias and conduction issues [159]. The body's regenerative capacity declines with age, limiting the heart's ability to repair post‐ischemia or injury, associated with a decrease in cardiac stem cell numbers and functionality, essential for tissue maintenance and repair [160].

MSC‐Exos have emerged as a therapeutic powerhouse in cardiovascular disease therapy, with capabilities that span from vascular regeneration to immunomodulation, inhibition of oxidative stress, and myocardial protection. These extracellular vesicles are equipped with a plethora of angiogenesis‐promoting growth factors, such as VEGF, FGF, and PDGF. These factors are instrumental in stimulating the formation of new blood vessels, thereby enhancing blood supply to damaged areas and improving microcirculation [154]. The miR‐let7/HMGA2/NF‐κB pathway is a key regulatory mechanism by which MSC‐Exos promote the polarisation of M2 macrophages within atherosclerotic plaques in mice, reducing atherosclerotic inflammation and contributing to the mitigation of cardiovascular disease [161]. Oxidative stress, a significant etiological factor in cardiomyocyte death, can be alleviated by miR‐129‐5p, a microRNA carried by MSC‐Exos. This molecule engages in the TRAF3/NF‐κB pathway, offering a promising approach to treating heart failure [162, 163]. Furthermore, MSC‐Exos have been shown to modulate lncRNA A2M‐AS1, which in turn regulates the miR‐556‐5p/XIAP axis in target cells. This regulation attenuates hypoxia/reoxygenation (H/R)‐induced oxidative stress in cardiomyocytes, providing a novel perspective on the protection of myocardial cells. Promotion of cardiomyocyte proliferation is pivotal in the treatment of acute myocardial infarction. Encapsulated within exosomes, hsa‐miR‐590‐3p downregulates genes such as Hopx that inhibit cell proliferation [164]. Once internalised by cardiomyocytes, these exosomes stimulate the proliferation of cells in the peri‐infarct region, aiding in the restoration of cardiac function [165]. Additionally, studies have demonstrated that bone marrow stem cells, under the influence of exosomes, can differentiate into cardiomyocytes when expressing GATA‐4. This process not only reduces cardiomyocyte apoptosis but also enhances myocardial function post‐infarction [166].

### 4.4 Mesenchymal Stem Cell‐Derived Exosomes in Neurodegenerative Diseases

Aging is the key risk factor for AD, a neurodegenerative disorder causing cognitive decline and memory loss. Evidence points to DNA damage and impaired repair mechanisms as significant contributors to AD [167]. Oxidative stress from ROS in high‐metabolic neurons can damage DNA. The capacity for base excision repair, vital for DNA damage recognition and repair, is reduced in mild cognitive impairment, indicating a decline in the brain's integrity with age [168]. Excessive DNA damage can lead to mistranslation by DNA polymerase, genome errors, and neurodegeneration. Mitochondrial dysfunction, particularly impaired mitophagy, causes oxidative stress and energy deficits, disrupting cellular metabolism and contributing to neuronal dysfunction [169, 170]. Epigenetic changes, such as DNA methylation and histone modifications, impact gene expression, affecting the brain's resilience and repair capacity, and influencing synaptic plasticity, neuronal survival, and inflammation, crucial for cognitive function [171]. Additionally, aging is linked to a decrease in neurotrophic factors, reducing neuronal maintenance and repair, and increasing brain vulnerability to degeneration. The complex interaction of these factors—DNA damage, mitochondrial issues, epigenetic changes, and neurotrophic decline—may foster the development of AD.

MSC‐Exos have shown promise in promoting neurogenesis, curbing inflammation‐induced neuronal damage in the hippocampus, reducing oxidative stress, and leveraging the targeted drug delivery capabilities of exosomes, thereby playing a role in neurodegenerative diseases. BMSC‐Exos have been demonstrated to stimulate neurogenesis and improve cognitive function by enhancing astrocyte formation and synaptic connectivity [172]. In the context of neurological disorders, ADSC‐Exos have been shown to promote HT22 cell proliferation and facilitate the repair of damaged neurons [173]. Moreover, miRNA‐146 and miRNA‐21 encapsulated within hUCMSC‐Exos and BMSC‐Exos are capable of polarising microglia towards an anti‐inflammatory M2 phenotype, thereby mitigating neuroinflammation and improving cognitive function through immunomodulation [174]. The expression of inflammatory mediators, including IL‐1β, IL‐6, TNF‐α, amyloid‐β1‐42 (Aβ1‐42), and phosphorylated tau (p‐Tau), in hippocampal microglia and astrocytes is suppressed, further alleviating neuroinflammation and cognitive dysfunction [175]. Trophoblast stem cell‐derived exosomes may reduce ROS production and oxidative stress by delivering miR‐100‐5p, which downregulates the expression of NOX4, thereby ameliorating dopaminergic neuronal injury in Parkinson's disease model mice through the NOX4‐ROS‐Nrf2 pathway [176]. Furthermore, exosomes can effectively traverse the blood–brain barrier, enabling distribution within the central nervous system and facilitating targeted drug delivery for neurodegenerative diseases. Jahangard et al. injected miRNA‐29 encapsulated within MSC‐Exos into an AD rat model and reported upregulation of miRNA‐29 in hippocampal samples, with downregulation of BIM and BACE1 proteins and reduced Aβ peptide levels, effectively ameliorating Alzheimer's symptoms [177]. Additionally, the surface of MSC‐Exos can be modified to enhance their targeting capabilities. The incorporation of CNS‐associated rabies virus glycoprotein peptides into the membranes of MSC‐Exos increases their targeting and aggregation in the hippocampus and cortical layers of AD animal models, offering a promising therapeutic strategy [178].

## 5 Prospect of Future Clinical Application

MSC‐Exos are transitioning from bench to bedside, with numerous clinical applications beginning to emerge (Table 3). MSC‐Exos show promise in addressing aging and age‐related diseases, but challenges remain. Sourcing MSCs varies in ethical considerations and technical hurdles persist in isolating MSC‐Exos, requiring improved purification methods to ensure clinical purity and consistency. Clinical use is hindered by the lack of standardised dosing and guidelines, necessitating rigorous trials to establish optimal regimens. The combination of exosome technology with cell‐penetrating peptides (CPPs) offers a new approach to drug delivery, potentially enhancing therapeutic precision and efficacy while mitigating side effects. This strategy could improve drug stability and bioavailability, marking a significant advancement in therapeutics. Direct secretion of bioactive factors from MSCs, independent of exosomal encapsulation, also plays a role in anti‐aging effects. As MSC‐Exos move towards clinical applications, they are showing potential in wound healing, bone regeneration, and neurodegenerative disease treatment, indicating a broad impact on medical care. However, the field requires careful development to realise these benefits fully.

**TABLE 3:** Clinical trials of MSC‐Exos in the clinical treatment.

Disease	Delivered	Process	Efficacy	Clinical trial number
ADSC‐Exos				
Alzheimer's disease	Nasal spray	Nine subjects aged ≥ 50 with AD received allogeneic ASC‐Exos	Primary endpoints included liver and kidney function, ADAS‐cog, and ADCS‐ADL scores within 3 months	NCT04388982
Periodontitis	Injection	10 subjects received local MSC‐Exos in periodontal pockets, followed for 6 months	Primary endpoints include changes in gingival inflammation, periodontal pocket depth, attachment level, and bone density	NCT04270006
Acne scars	Epidermal application	25 volunteers, aged 19–54, treated with ASC‐Exos combined with CO2 FCL	The treated side demonstrated reduced pore size and skin surface roughness from baseline	[179]
BMSC‐Exos				
Epidermolysis bullosa	Wounds application	6 doses of BM‐MSC‐Exos over 3 months	Primary endpoint: dose‐limiting toxicity; secondary endpoint: wound size	NCT04173650
Severe COVID‐19	Intravenous	Dose of 1–10 × 10^6 MSCs/kg	Survival rate of 83%; approximately 71% of patients achieved clinical recovery, 13% remained critically ill but stable, and 16% died from unrelated causes. Clinical status and oxygenation improved, with reductions in C‐reactive protein by 77%, ferritin by 43%, and D‐dimer by 42% following a single dose	NCT04276987
Knee Osteoarthritis	Injection	33 veterans received a single 2 mL dose of Exo	Significant improvements in Brief Pain Inventory and Oswestry Disability Index scores by 77% and 80% at 6 months. Upper and lower extremity function scores improved by 51% and 76%, respectively	NCT05060107
Stroke	Injection	5 subjects aged 40–80 received a single 200 mg dose of miR‐124‐transfected BMSC‐Exos in the ischemic area 1 month post‐stroke	12‐month follow‐up for recurrent stroke, cerebral edema, seizures, and ischemic‐to‐hemorrhagic transformation. Improvement assessed using the Rankin scale	NCT03384433
hUCMSC‐Exos				
Chronic GVHD‐related Dry Eye	Artificial tear drops	27 subjects aged 18–70 used artificial tears for 2 weeks, then 10 mg UC‐MSC‐Exos four times daily for 2 weeks	Primary endpoint: change in Ocular Surface Disease Index score at 12 weeks; secondary endpoints: tear production, tear film breakup time, fluorescein staining area, eye redness, tear meniscus, and best‐corrected visual acuity	NCT04213248
Type 1 diabetes mellitus	Intravenous injection	20 patients aged 18–60 with C‐peptide reduction > 50%, C‐peptide ≥ 0.8 ng/mL, insulin ≥ 0.4 IU/kg/d received two consecutive intravenous injections	Exosomes characterised by CD63, CD9, Alix, TSG101, HSP70; MVs by Annexin V, Flotillin‐2, selectins, integrins, CD40 metalloprotease. Primary endpoints: changes in insulin dose and HbA1c levels	NCT02138331
Chronic kidney disease	Intravenous and arterial injection	40 patients with CKD stage III or IV, aged 26–44, randomised 1:1 into a control group (saline) or UCB‐MSC‐Exos group (100 μg/kg)	Significant improvements in glomerular filtration rate, serum creatinine levels, blood urea, and urinary albumin/creatinine ratio in the UCB‐MSC‐Exos group	[29]
Allogeneic MSC‐Exos				
Pulmonary infection	Aerosol inhalation	60 subjects aged 18–75 randomised to low‐dose, high‐dose, or placebo groups	Low and high‐dose groups received 7 doses of 8.0 × 10^8 ASC‐Exos/3 mL and 16.0 × 10^8 ASC‐Exos/3 mL, respectively	NCT04544215
Acute respiratory distress syndrome	Aerosol inhalation	Subjects randomised to low, medium, and high‐dose groups receiving 2.0 × 10^8, 8.0 × 10^8, and 16.0 × 10^8 MSC‐Exos daily for 1 week	Significantly enhances the survival rate of ARDS induced by SARS‐CoV‐2	NCT04602104
MSC‐Exos				
Pancreatic cancer	Intravenous injection	28 adults aged ≥ 18 received MSC‐Exos on days 1, 4, and 10, repeated every 14 days for up to 3 cycles	Aim to determine maximum tolerated dose and dose‐limiting toxicity	NCT03608631
Macular degeneration	Local injection	44 patients with stage 1 macular hole received 50 or 20 μg MSC‐Exos, followed for 6 months	Efficacy assessed by minimum linear diameter of the hole measured by OCT at 24 weeks post‐surgery	NCT03437759
COVID‐19	Inhalation	30 patients aged 18–65 received 3 mL of MSC‐Exos twice daily for 10 days	Primary endpoint: adverse events 30 days post‐discharge	NCT04491240

## 6 Conclusion

In this review, we have delved into the complex mechanisms underlying the anti‐aging effects of MSC‐Exos and their burgeoning application in age‐related diseases. As our understanding of these mechanisms advances, the therapeutic use of MSC‐Exos is poised to become more precise and widely integrated into clinical practice. This deeper insight is paving the way for the development of more effective treatment strategies.

In conclusion, MSC‐Exos represent a highly promising and potent therapeutic approach for a variety of age‐related conditions. As we advance in the development and manufacturing of MSC‐Exos, it is imperative to adhere to stringent ethical standards. This commitment involves ensuring informed consent for stem cell sourcing, transparency in research, and maintaining rigorous safety and efficacy in clinical use. By integrating these ethical considerations, MSC‐Exos can be responsibly and effectively incorporated into medical practice, offering hope for a future where age‐related diseases are managed with unprecedented efficacy and respect for individual dignity. The scientific community looks forward to a forthcoming wave of rigorous research.

## Author Contributions

**Bohua Wei:** writing – original draft, methodology, conceptualization, visualisation. **Mengting Wei:** writing – original draft, conceptualization, visualisation. **Haonan Huang:** writing – original draft, formal analysis. **Ting Fan:** writing – review and editing, formal analysis, software. **Zhichang Zhang:** writing – review and editing, supervision, software, funding acquisition, data curation, conceptualization. **Xiaoyu Song:** writing – review and editing, funding acquisition, conceptualization, project administration, supervision.

## Ethics Statement

The authors have nothing to report.

## Consent

No written consent has been obtained from the patients as there is no patient identifiable data included.

## Conflicts of Interest

The authors declare no conflicts of interest.

## Data Availability

Data sharing not applicable to this article as no datasets were generated or analysed during the current study.
